# Supplementary material for: High-performance p-channel transistors with transparent Zn doped-CuI
Source: Nat Commun. 2020 Aug 27;11:4309. doi: 10.1038/s41467-020-18006-6 (PMC7453006; doi:10.1038/s41467-020-18006-6)
Supplement: Supplementary file 1 — Supplementary information [file 41467_2020_18006_MOESM1_ESM.pdf]

Supplementary information

High-performance *p*-channel transistors with transparent Zn doped-CuI

By Ao Liu,[†] *Huihui Zhu,*[†] *Won-Tae Park, Se-Jun Kim, Hyungjun Kim, Myung-Gil Kim,* and Yong-Young Noh**

Prof. Y.-Y. Noh, A. Liu, H. H. Zhu,

Department of Chemical Engineering, Pohang University of Science and Technology, Pohang, Gyeongbuk, 37673, Republic of Korea

E-mail: yynoh@postech.ac.kr

Dr. W.-T. Park

Department of Electrical and Computer Engineering, University of Waterloo, 200 University Avenue West, Waterloo, ON, N2L, 3G1, Canada

Prof. H. Kim, S.-J. Kim,

Department of Chemistry, Korea Advanced Institute of Science and Technology, Daehak-ro 291, Yuseong-gu, Daejeon 34141, South Korea

Prof. M.-G. Kim

School of Advanced Materials Science & Engineering, Sungkyunkwan University, Suwon 16419, Republic of Korea

E-mail: myunggil@skku.edu

Keywords**:** inorganic p-type semiconductor; thin-film transistor; copper iodide; low-temperature solution process; high hole mobility

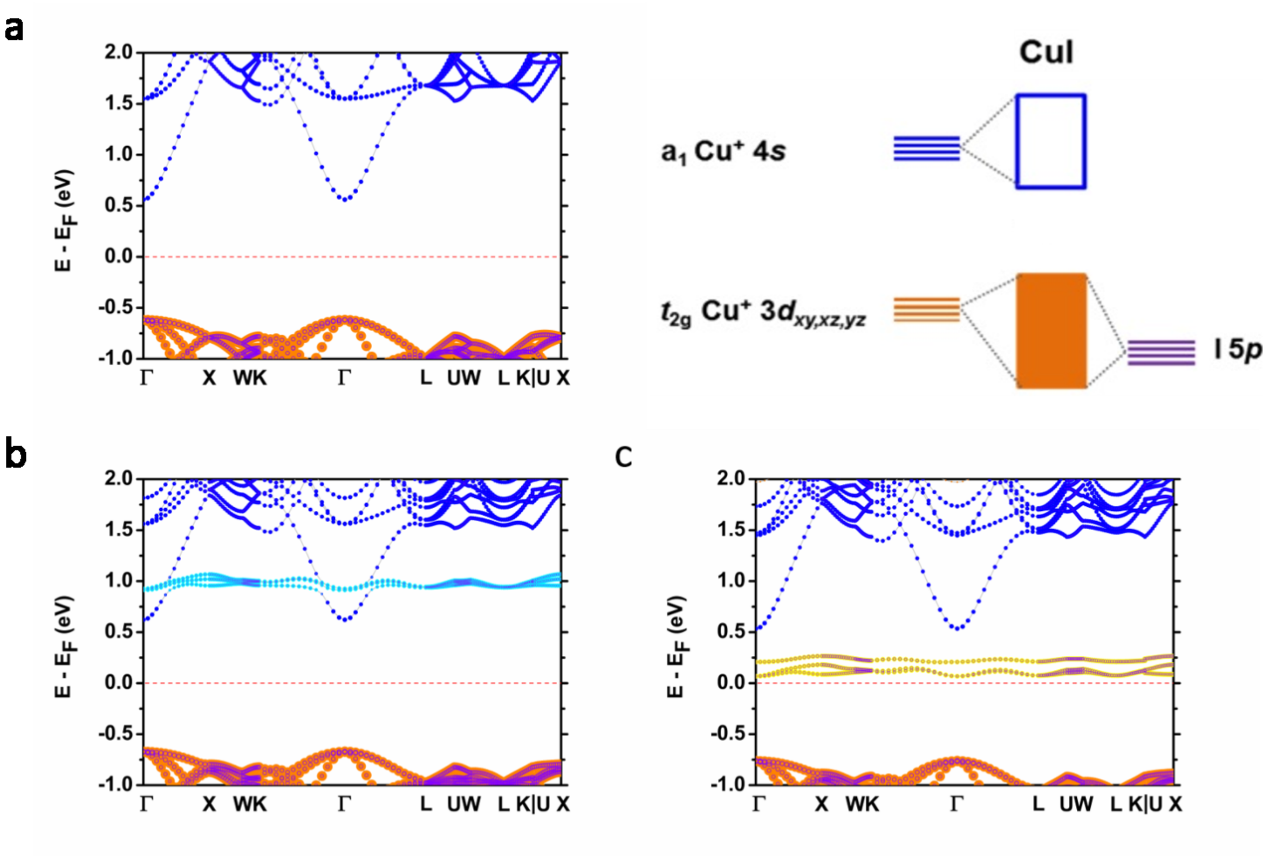

Supplementary Figure 1. (a) Projected band structure of defect-free CuI, where Cu 3*d* (orange), Cu 4*s* (blue), and I 5*p* (purple) states are shown. Corresponding schematic figure showing the orbital characteristics of VBM and CBM is also shown. (b) Projected band structure of CuI with 3% Cu vacancy and Pb^{2+} substitutional doping, where Cu 3*d* (orange), Cu 4*s* (blue), I 5*p* (purple), and Pb^{2+} (cyan) states are shown. (c) Projected band structure of CuI with 3% Cu vacancy and Bi^{3+} substitutional doping, where Cu 3*d* (orange), Cu 4*s* (blue), I 5*p* (purple), and Bi^{3+} (yellow) states are shown.

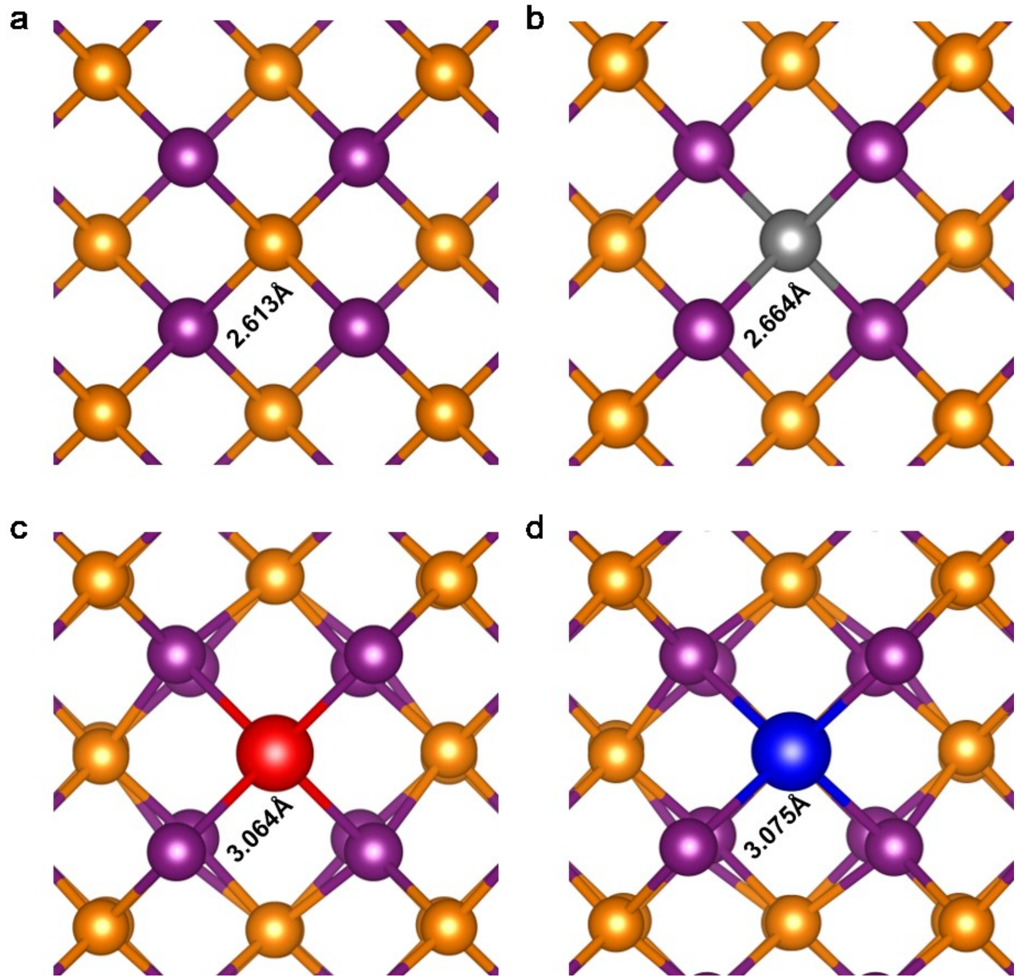

Supplementary Figure 2. DFT-optimized structures showing the local lattice distortion near the aliovalent doped sites; (a) defect-free CuI, (b) Zn^{2+}(gray)-doped CuI, (c) Pb^{2+}(red)-doped CuI, and (d) Bi^{3+}(blue)-doped CuI. Due to large size of Pb^{2+} and Bi^{3+}, a noticeable amount of lattice distortion is found.

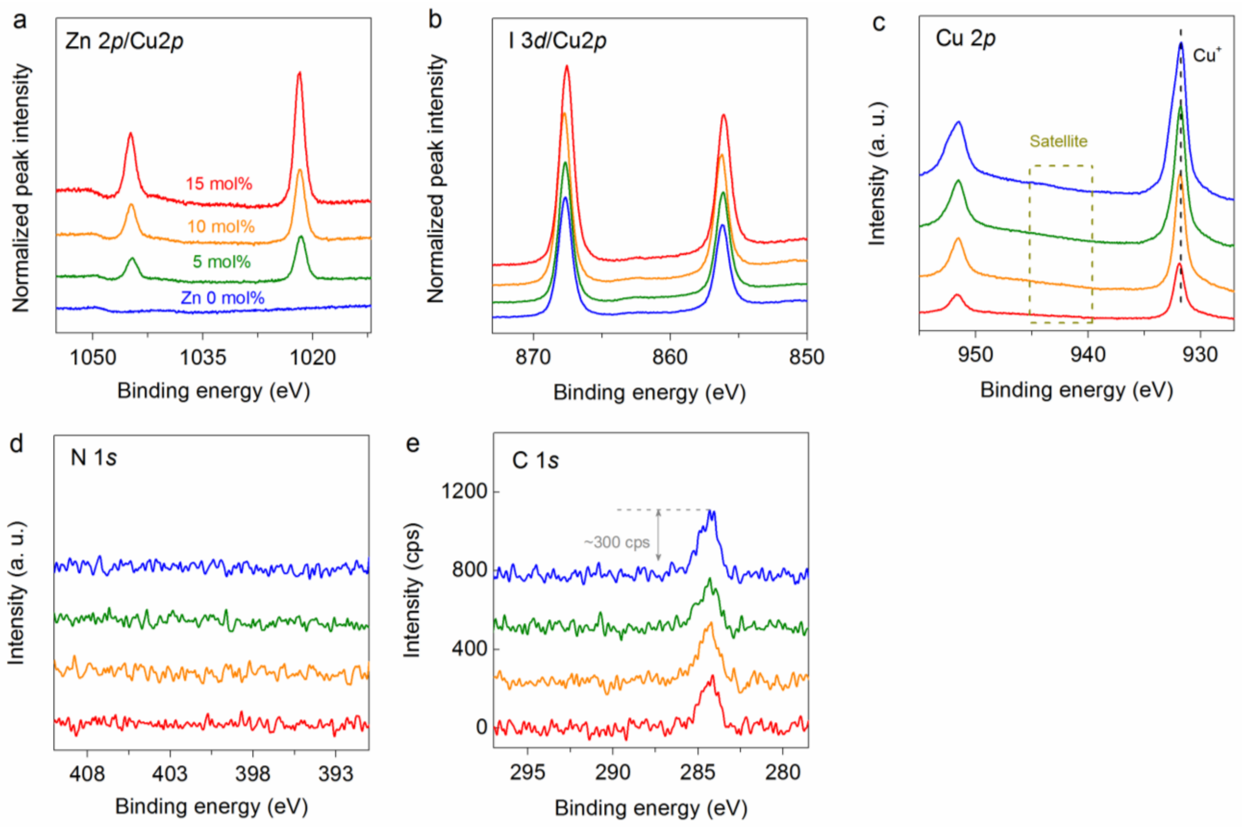

Supplementary Figure 3. X-ray photoelectron spectroscopy spectra of (a) normalized Zn 2*p*/Cu 2*p*, (b) I 3*d*/Cu 2*p*, (c) Cu 2*p*, (d) N 1*s*, and (e) C1*s* peaks for CuI:Zn thin films with different Zn^{2+} doping contents (0, 5, 10, 15 mol%).

The chemical components of different CuI:Zn thin films were clarified using X-ray photoelectron spectroscopy (XPS). As shown in Fig. 2c and supplementary Fig. 3a, the normalized Zn 2*p* peak intensity linearly increases with the Zn^{2+} content, which confirms the increased dopant concentration. No satellite peak is observed between Cu 2*p* peaks, indicating that only Cu^{+} exists in the CuI:Zn films. The N signal cannot be detected for all samples. As for C 1*s* spectra, only one single peak at ~284.3 eV was observed with low intensity of ~300 cps, indicating carbon mainly originates from the adsorbed adventitious contaminants on film surface.[1]

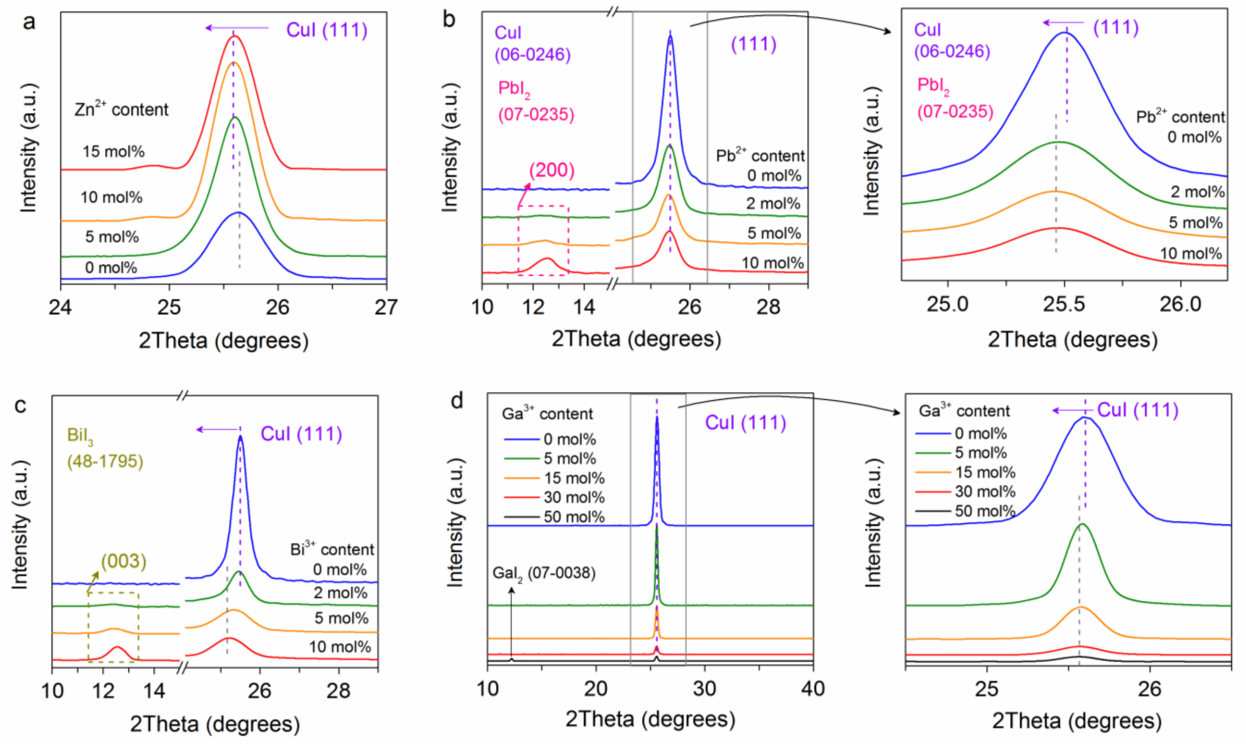

Supplementary Figure 4. XRD patterns of (a) CuI:Zn, (b) CuI:Pb, (c) CuI:Bi, and (d) CuI:Ga thin films with different dopant contents.

For pristine CuI, the strong diffraction peak located at 2θ = 25.5° is assigned to CuI (111) plane (JCPDS card No. 06-0246). The peak intensity is significantly decreased after Pb^{2+} or Bi^{3+} doping, which means the CuI film crystallinity was effectively suppressed. Due to the larger ionic radii of Bi^{3+} (108 pm) and Pb^{2+} (120 pm) compared to those of Cu^{+} (96 pm), the substitution at Cu sites leads to an increased lattice constant, resulting in the diffraction peak shifts toward lower angles. The smaller shift caused by the addition of Pb^{2+} can be understood by the low doping efficiency. Although the Ga^{3+} (62 pm) have a smaller size than Cu^{+}, the Ga^{3+} doping in CuI slightly shifts the CuI (111) plane towards a lower diffraction angle. This effect can be also understood by the Cu vacancies getting filled by Ga^{3+} leading to a slightly expanded lattice. However, owing to the limited solubility in CuI matrix (large ionic radii), separated PbI_2 and BiI_3 phases appear at lower angle and the intensities increase with higher doping contents. As for the Ga^{3+} doped CuI, the slightly shift of CuI (111) plane shift toward lower diffraction angle was also observed, which can be understood by the Ga^{3+} filling on Cu vacancies and thus the slightly expanded lattice.

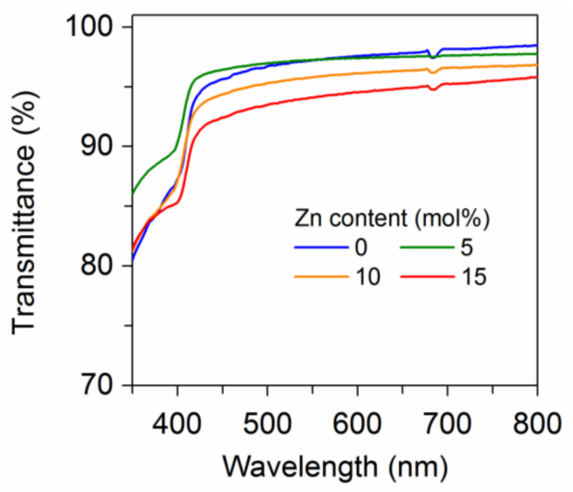

Supplementary Figure 5. Optical transmittances of CuI thin films doped with different amounts of Zn^{2+} (0, 5, 10, and 15 mol%).

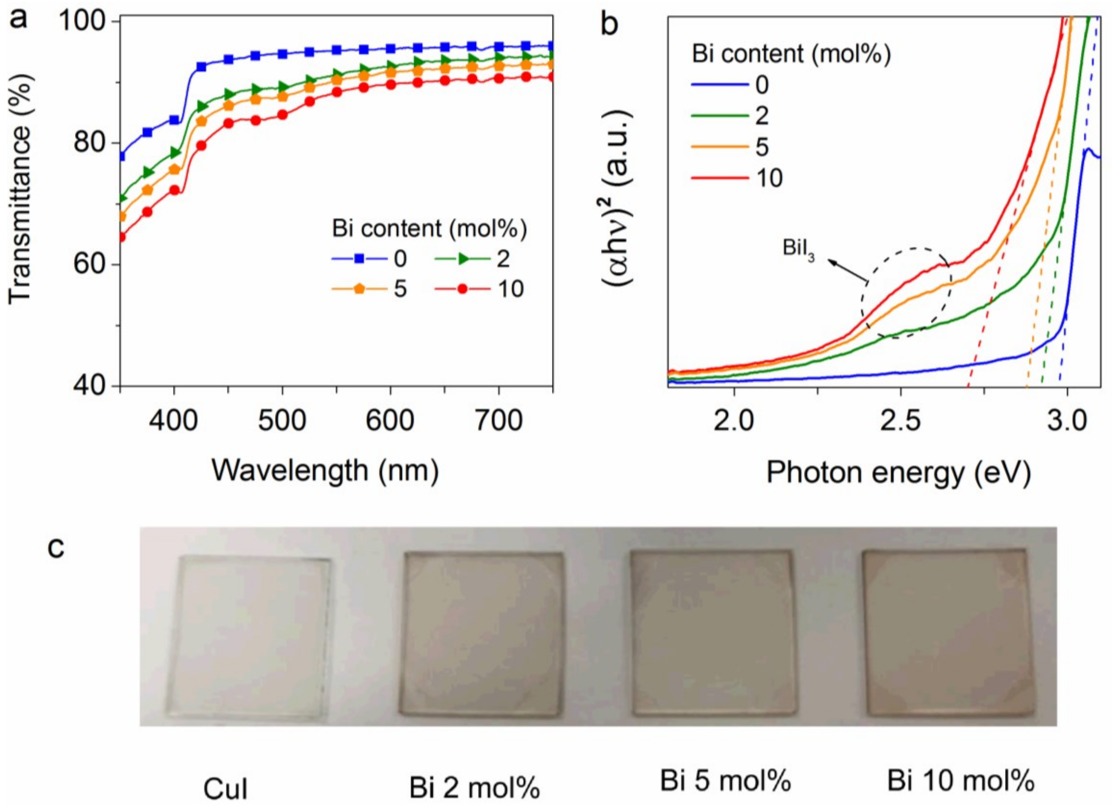

Supplementary Figure 6. (a) Optical transmittances, (b) Tauc plots, and (c) photographs for the CuI:Bi thin films doped with different Bi contents (0, 2, 5, and 10 mol%).

For Bi-doped CuI thin films, the optical transmittance and bandgap values reduced remarkably with increasing doping content. In addition, the obvious extra absorption was noted in the $(\alpha h\nu)^2$ to $h\nu$ curves, which could be attributed to segregated BiI_3 phase. This also implies the poor doping efficiency of Bi^{3+} in CuI matrix. For the CuI thin films incorporated with other dopants (*e.g.*, Ni^{2+}, Pb^{2+}, and Sn^{4+}), the optical transmittance reduced significantly, just like Bi-doped ones (data not shown here).

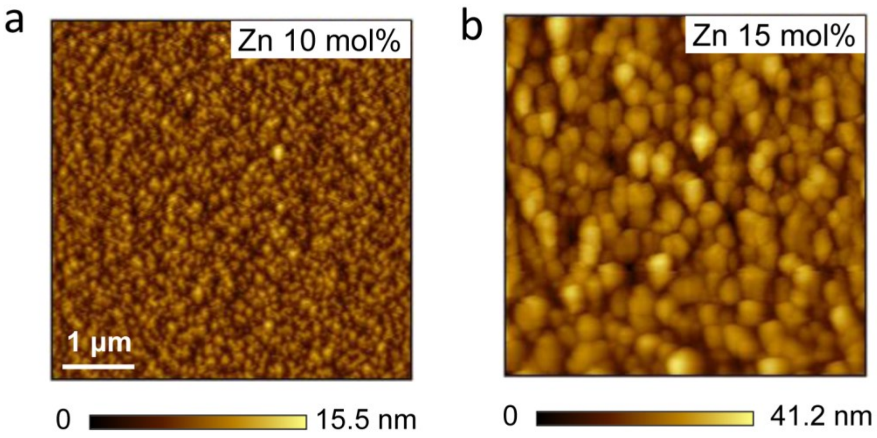

Supplementary Figure 7. AFM images of the CuI:Zn thin films doped with (a) 10 mol% and (b) 15 mol% Zn^{2+}.

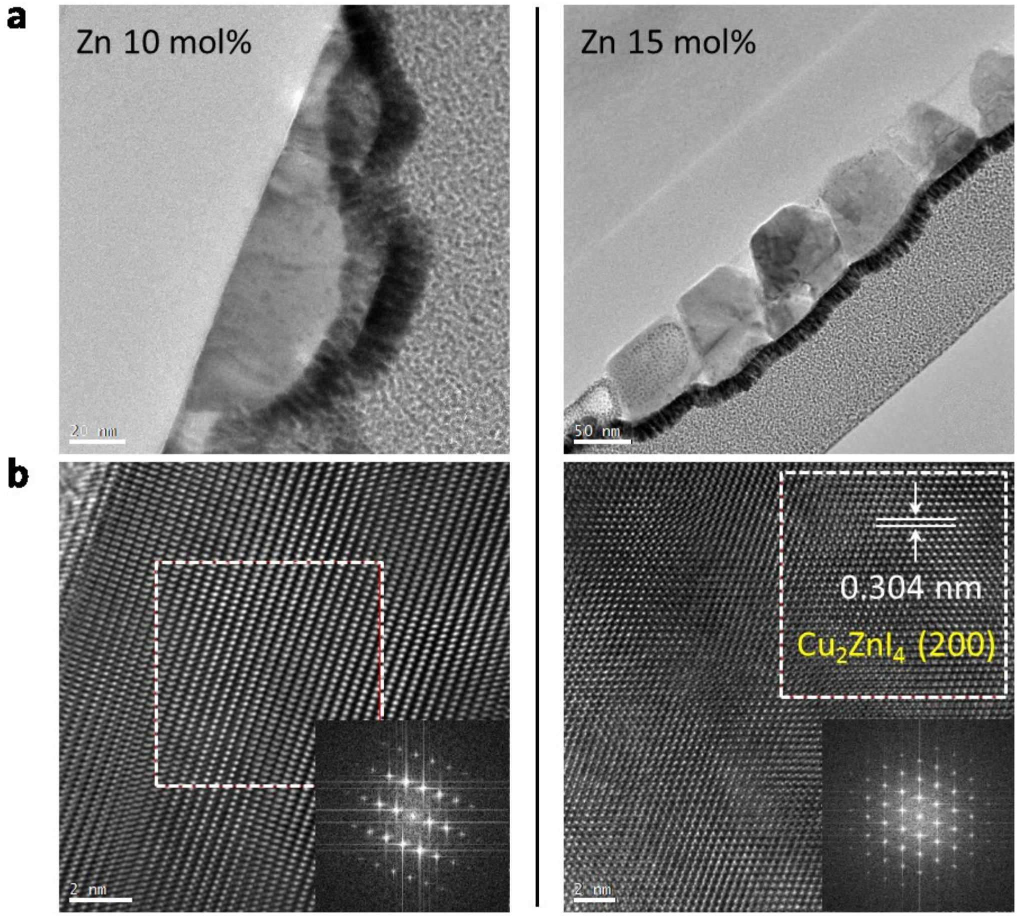

Supplementary Figure 8. (a) TEM and (b) HRTEM and FFT images of CuI:Zn films with various Zn doping contents (10, 15 mol%).

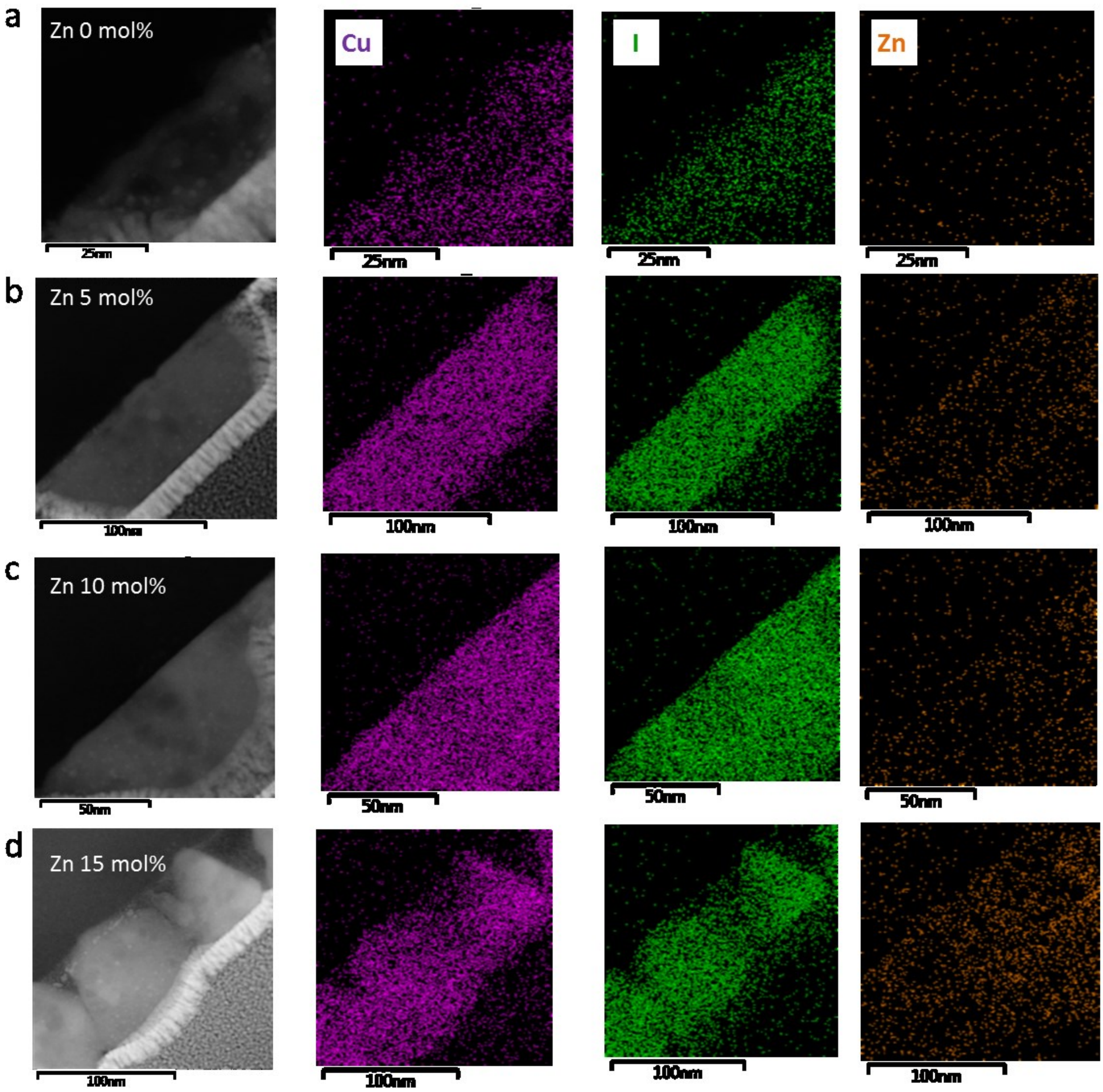

Supplementary Figure 9. Energy-dispersive X-ray spectroscopy mapping images of different CuI:Zn samples with variation of Zn doping contents (0, 5, 10, 15 mol%).

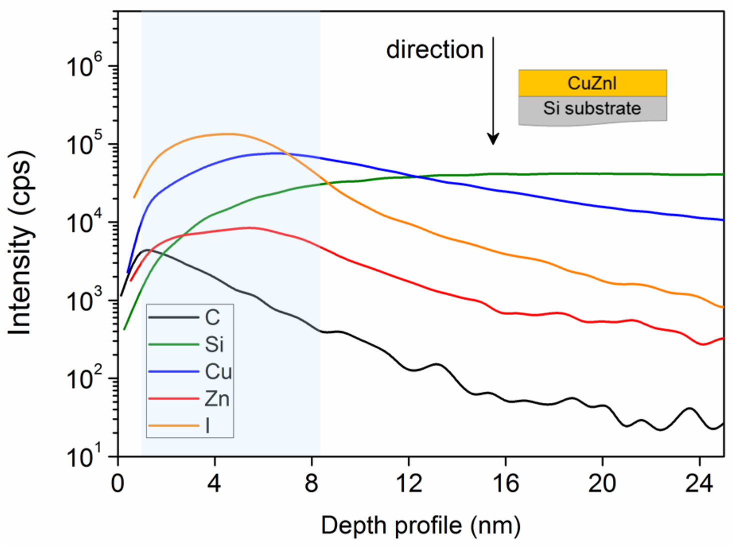

Supplementary Figure 10. SIMS spectra of element distribution in CuI:Zn thin film.

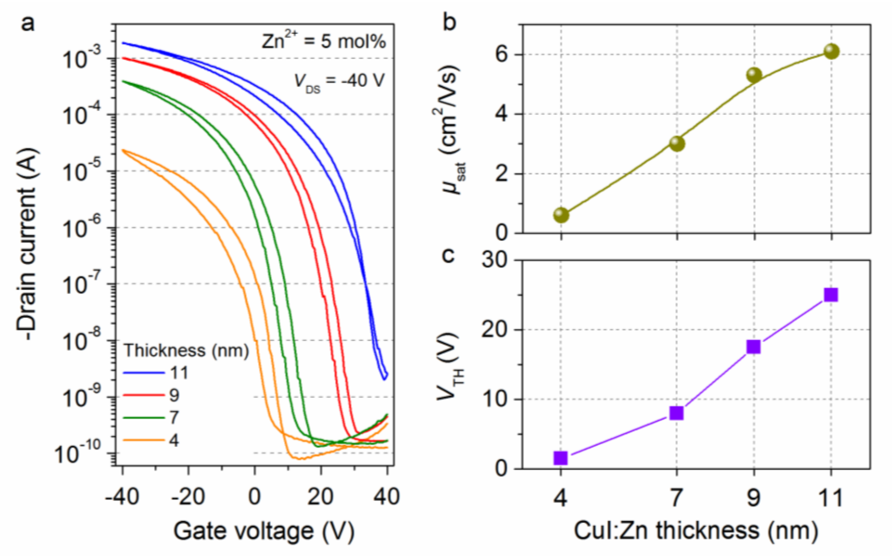

Supplementary Figure 11. (a) Transfer curves of CuI:Zn TFTs (Zn^{2+} = 5 mol%) as a function of channel thickness. Summarizations of (b) μ_{sat} and (c) V_{TH} of corresponding devices.

The optimisation of channel thickness is important to achieve high performance TFTs. Due to the high hole concentration in CuI:Zn channel layers, their thickness should be thinned to deplete excessive holes for the high I_{on}/I_{off}. We investigated the thickness-dependent device performance through adjusting CuI solution concentration while fixing the Zn^{2+} doping amount, i.e., 5 mol%. The monotonically decreased μ_{sat} and negative V_{TH} shift along with the thinner film thickness could be mainly attributed to the reduced absolute hole amounts, which is similar to our previous observation on CuI TFTs[2].

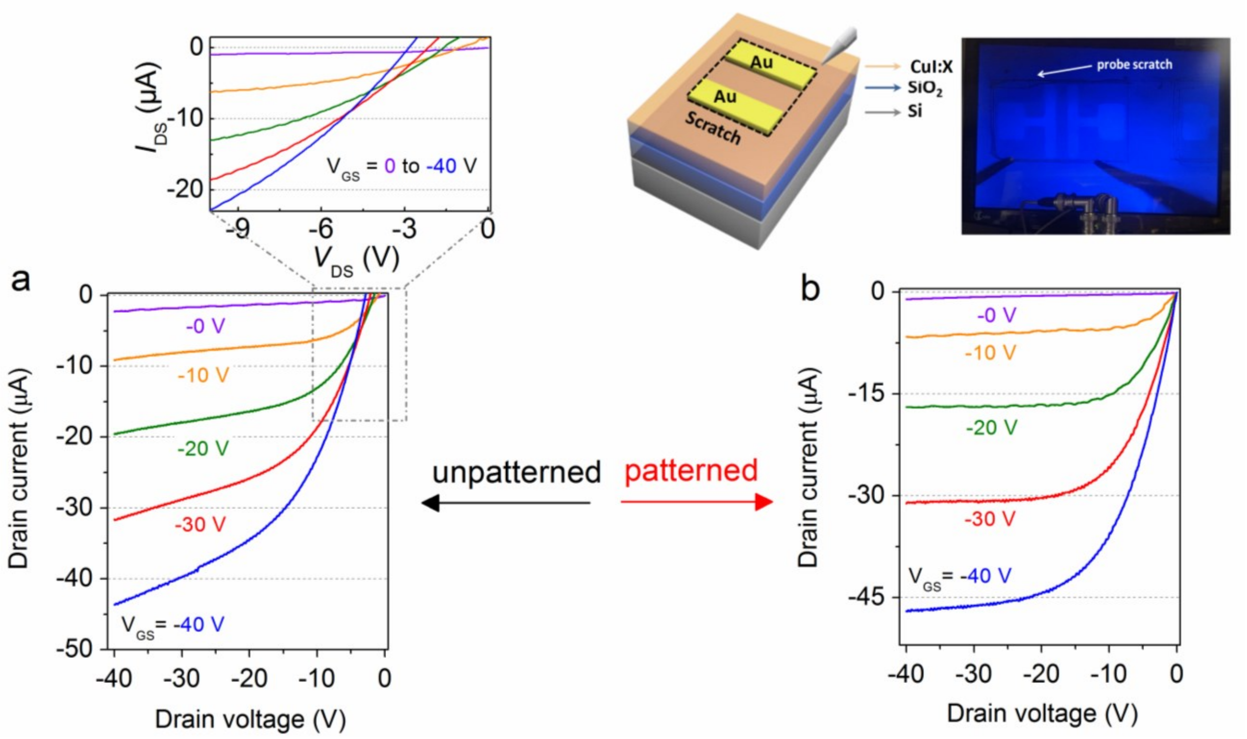

Supplementary Figure 12. Output curves for the (a) unpatterned and (b) patterned TFTs. The figure above (b) describes the probe tip ablation process and the patterned channel image.

In the case of the unpatterned channel configuration, the TFTs always deliver high gate leakage currents, especially under large negative gate voltages (V_{GS}). As shown in supplementary Fig. 12a, I_g results in the distortion of the output curves in the linear region and poor current saturation in the saturation region. To pattern CuI:X channel layers, the probe tip ablation method[3] was used instead of the conventional photolithographic process because the film's electrical property could be degraded during the photolithography process. Different from metal-oxide materials, the hardness of metal-halide films is quite low because of the M^{n+} and I^- ions stacking and the weak Columbic interaction. As shown in supplementary Fig. 12b, the "patterned" device affords more ideal output curves with a clear pinch-off and current saturation.

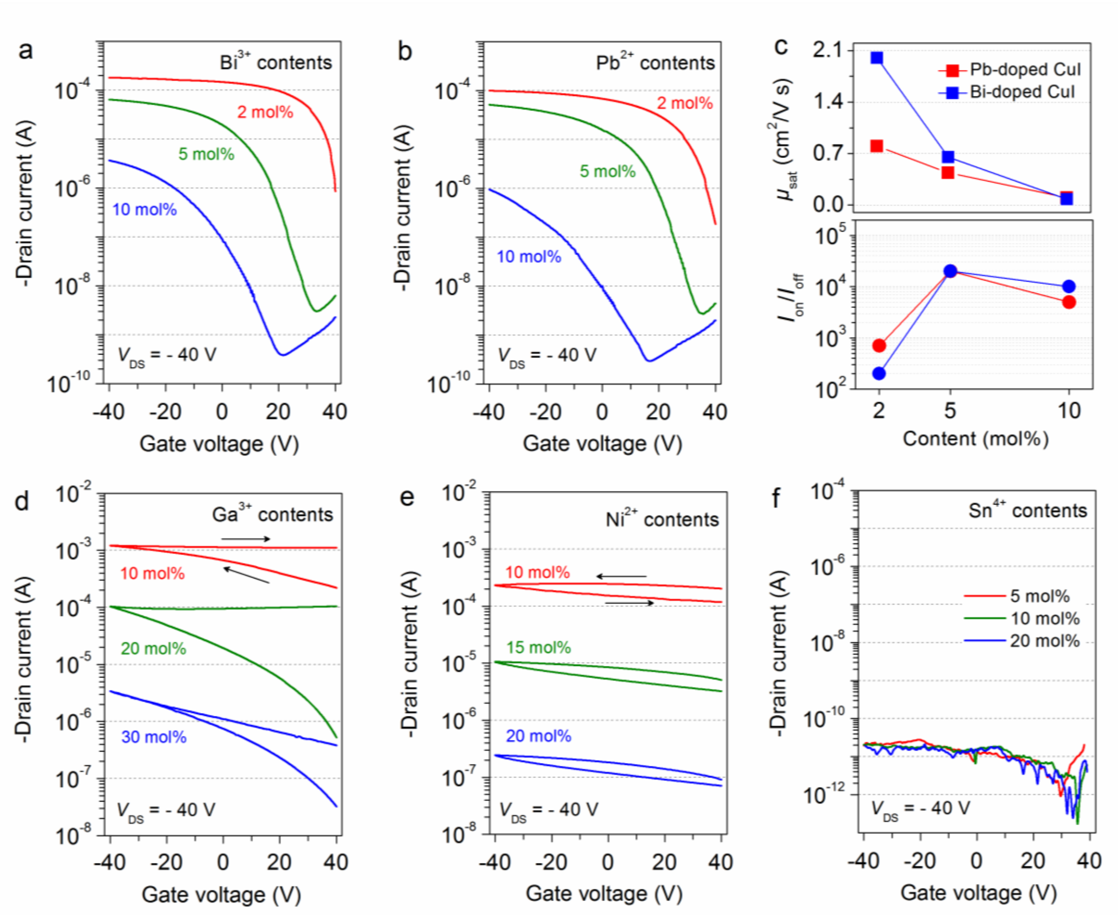

Supplementary Figure 13. Transfer characteristics of CuI TFTs doped with different dopants (M^{n+} = Pb^{2+}, Bi^{3+}, Sn^{4+}, Ga^{3+}, and Ni^{2+}).

The μ_{sat} of CuI:Bi$_{5\ mol\%}$ TFTs is 0.45 cm^2 V^{-1} s^{-1}, which is slightly higher than those of Pb-doped devices (0.34 cm^2 V^{-1} s^{-1} with 5 mol% Pb^{2+}). Given a larger ion radius of Pb^{2+} (120 pm) than Bi^{3+} (108 pm), the lower doping efficiency is expected. Despite the notable current inhibition for Ga^{3+}-doped CuI TFTs, all the devices exhibit poor current modulation behavior without reliable OFF state. The small ionic radius of Ga^{3+} makes it prefer to act as interstitial impurities rather than fill in copper vacancies or substitute Cu^{+}. The statement is confirmed by the clockwise hysteresis in double-sweep transfer curves, revealing that mobile impurity ions exist in the CuI:Ga channel layer.[4] As for the Ni^{2+}-doped devices, no ideal transistor was observed. Considering the improper octahedral geometry and facile hydrate formation tendency in NiI_2, the results indicate that Ni^{2+} is not suitable as CuI dopant. We also tried Sb^{3+} and In^{3+} as dopants in CuI matrix. However, it is unsuccessful to prepare the mixed solution no matter in air or inert environment.

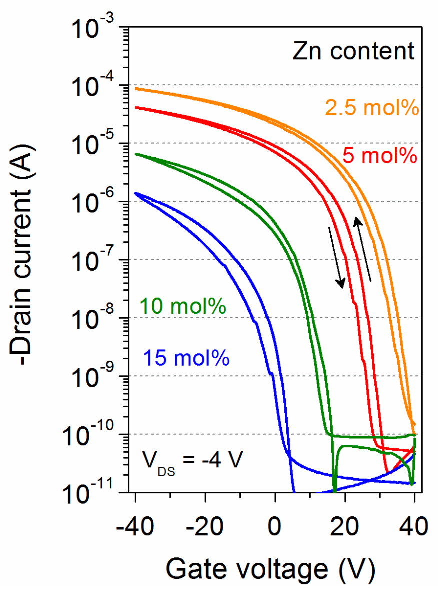

Supplementary Figure 14. Transfer curves of Zn-doped CuI TFTs with different Zn^{2+} contents in the leaner region (V_{DS} = -4 V). The corresponding μ_{lin} values are 5.1, 4.5, 1.3, and 0.25 cm^2/Vs for the 2.5, 5, 10, and 15 mol% Zn-doped CuI TFTs, respectively.

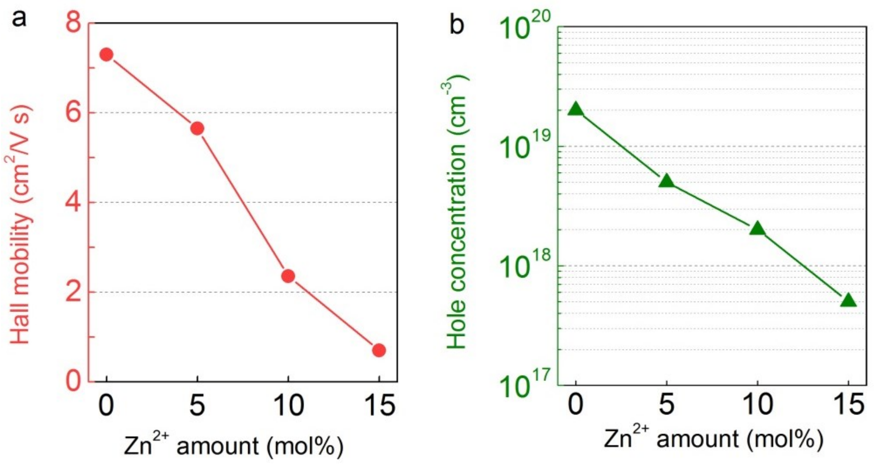

Supplementary Figure 15. (a) Hall mobility and (b) hole concentration of CuI:Zn thin films as a function of Zn^{2+} doping content.

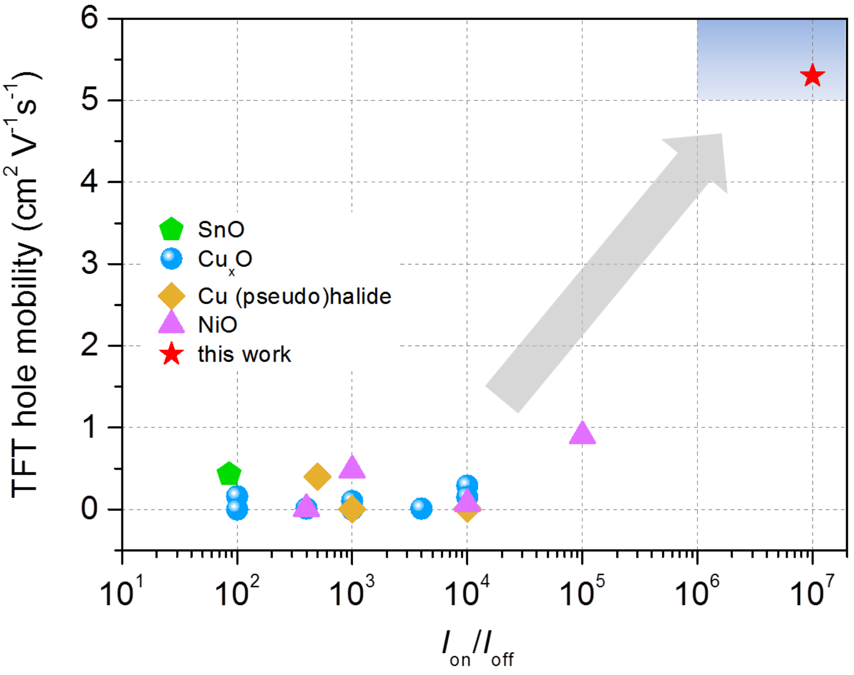

Supplementary Figure 16. Benchmark of hole mobility and I_{on}/I_{off} for reported solution-processed inorganic p-channel TFTs on SiO_2 dielectric. The extracted data are listed in Supplementary Table 3.

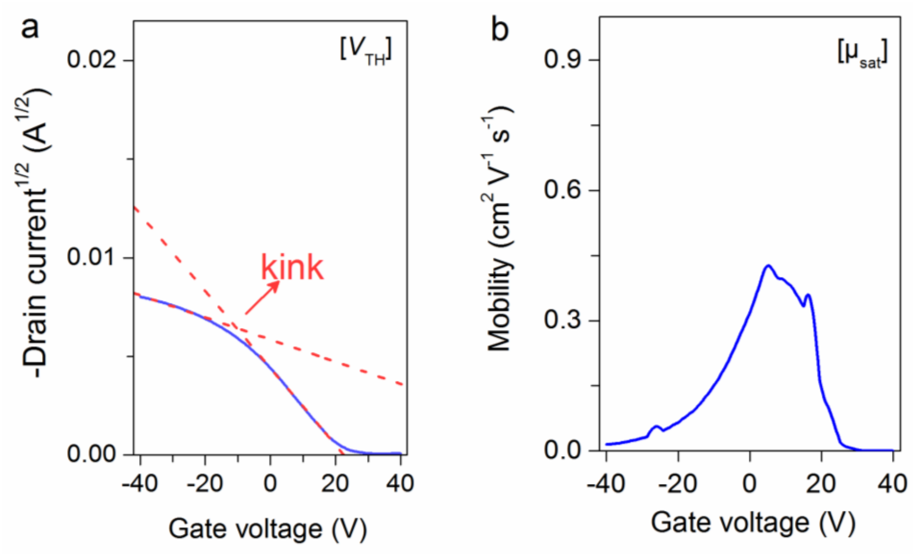

Supplementary Figure 17. (a) V_G *vs*. $I_{DS}^{1/2}$ and (b) V_G *vs*. mobility curve of 5 mol% Bi-doped CuI TFTs.

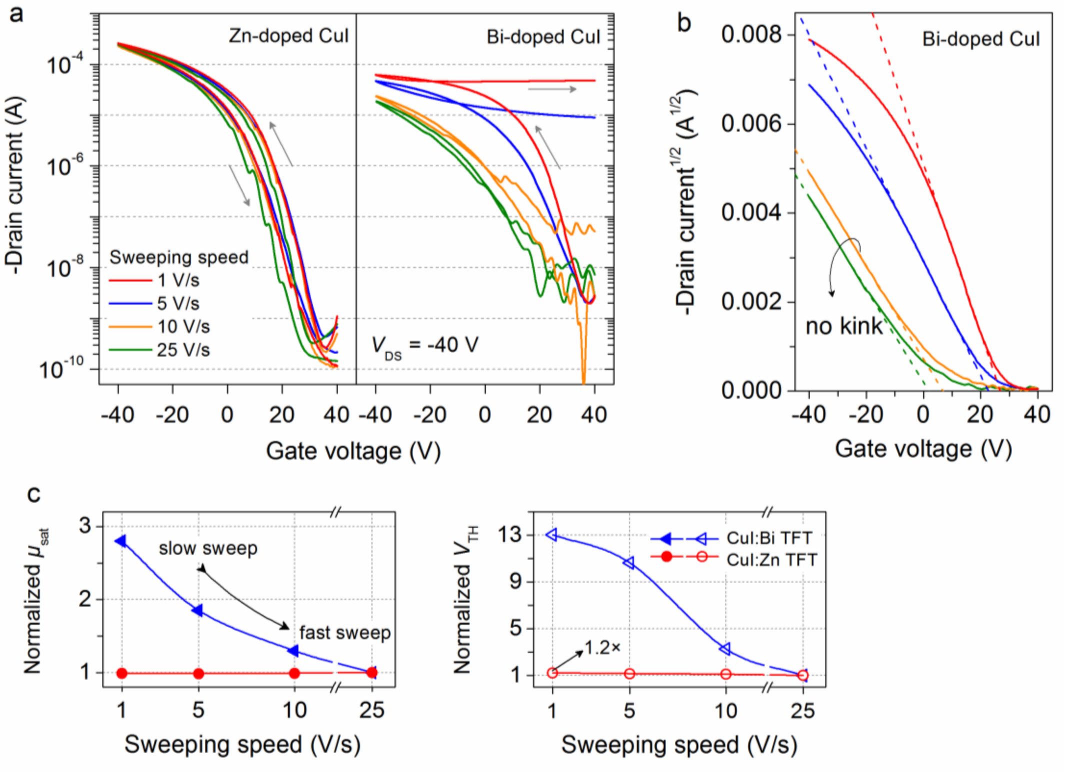

Supplementary Figure 18. (a) Transfer characteristics of Zn- and Bi-doped CuI TFTs with different sweeping speeds. (b) Sqrt (drain current) *vs.* gate voltage curves for CuI:Bi TFT as a function of scan speed. (c) Summarizations of normalized μ_{sat} and V_{TH} for the TFTs measured under different scanning speeds.

Due to a low doping efficiency for Bi^{3+} in CuI matrix, a certain amount of mobile ions existed in the film. In addition, the Bi^{3+} addition dramatically deteriorated CuI crystallinity (Figure S4c) and thus the formation of more grain boundaries. Several recent studies have revealed that the grain boundaries can serve as effective paths for ion migration in polycrystalline perovskite films. The low-speed scanning enables the contributions of more ion conduction and thus higher current level. Meanwhile, the reverse sweep of transfer curves under low scan speed has no effective field-effect modulation behavior, which indicated the ionic conduction dominated rather than electronic (hole) transportation.

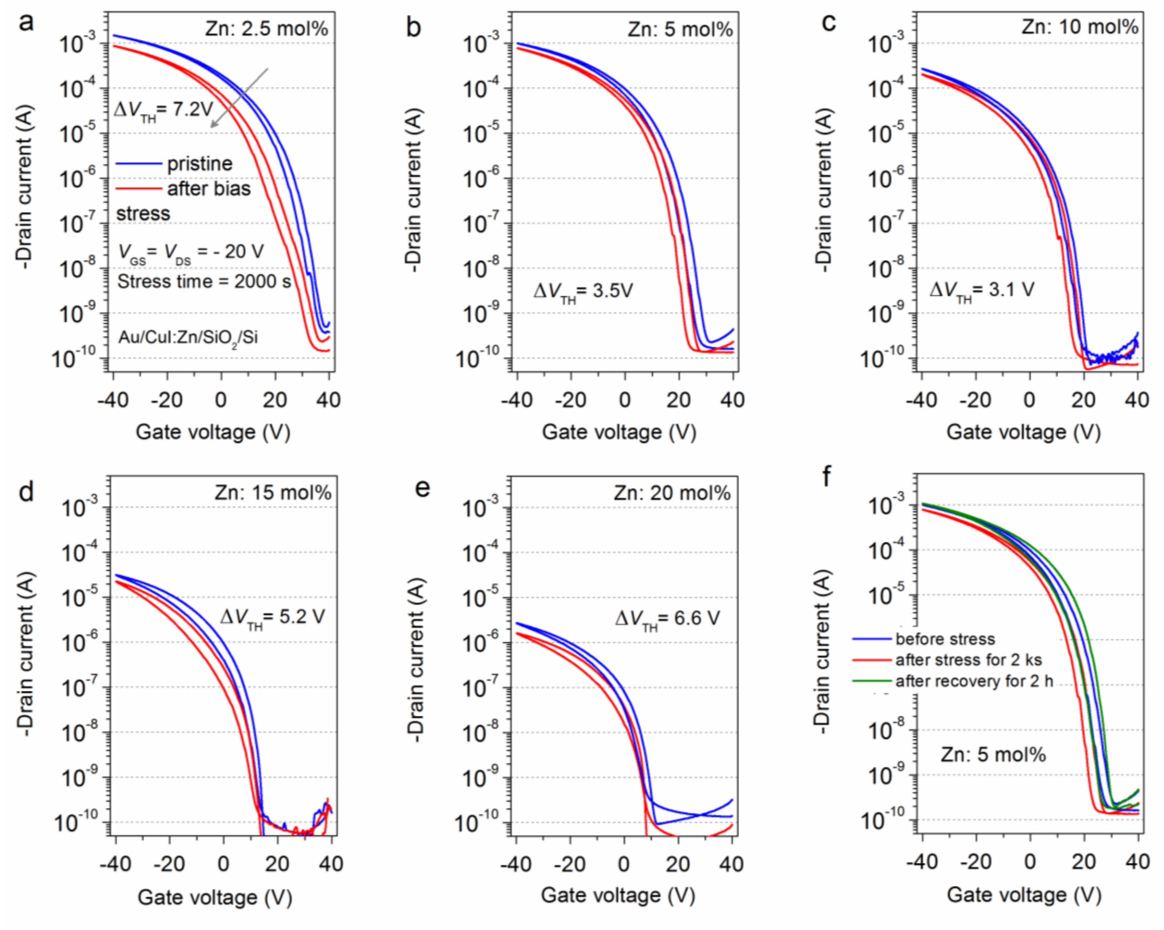

Supplementary Figure 19. (a-e) Transfer curves of different CuI:Zn TFTs before/after negative bias stress tests. (f) Transfer curve shifts during the recovery process after terminating the applied voltage (CuI:$Zn_{5mol\%}$ TFT).

The subthreshold slope remained relatively stable under bias stress test, indicating that the creation of extra defect states at CuI:Zn/SiO_2 interface and/or in the CuI:Zn channel is negligible, and charge trapping is the dominant instability mechanism.

Figure 19f shows the transfer curve recovery behavior after terminating the applied voltages (V_{GS} = V_{DS} = 0 V). The transfer curve shifted toward a positive direction to the original one, which can be attributed to the hole de-trapping from the interface or bulk dielectric to the active layer.

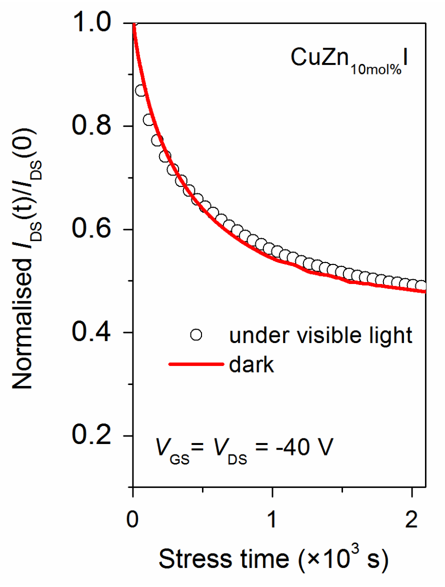

Supplementary Figure 20. Negative bias stress-induce I_{DS} variations of CuI:Zn TFTs under dark and visible light (jcr 15v-150wbn halogen lamp with 150 Watt power and 590 nm wavelength light source).

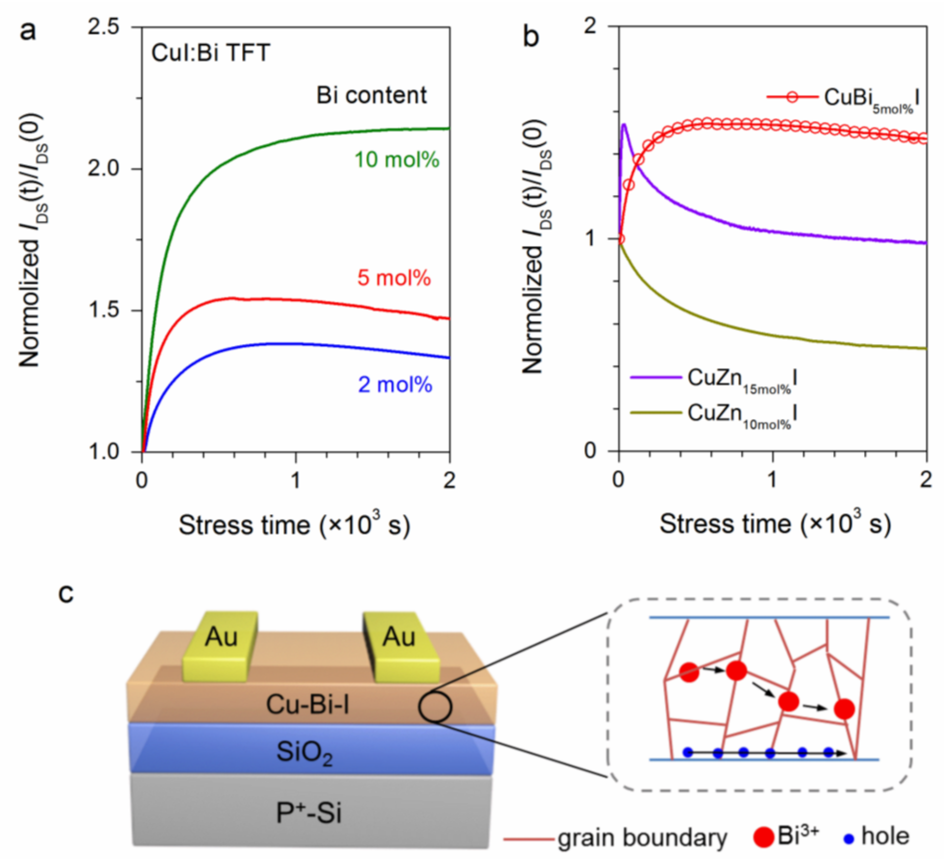

Supplementary Figure 21. (a) Negative-bias-stress results for CuI TFTs doped with different Bi^{3+} contents (V_{DS} = V_{GS} = -40 V). (b) Stability comparison between Zn- and Bi-doped CuI TFTs. (c) Schematic description of ion migration along grain boundaries in CuBiI channel under bias voltage.

For CuI:Bi TFTs, all the devices exhibited abnormal increased I_{DS} under negative bias stress test. Because of the large ionic radius of Bi^{3+} (109 pm) than Cu^{+} (77 pm), the doping efficiency of Bi^{3+} in CuI matrix is low. As a result, certain of mobile ions existed in the film. Under the constant gate-bias condition, the slow moving ions shifted to accumulation region and contribute the ion conduction. Actually, the ionic conduction and the charge trapping at dielectric/semiconductor interfaces are the two competing phenomenon occurring at the same time. With longer bias-stress time, the charge trapping became dominated and thus the drain current began to saturate or reduce. As for the Zn-doped CuI, due to high doping efficiency of Zn^{2+} and low valence state compared with Bi^{3+}, the polarization phenomenon occurred in a very short time even with high Zn^{2+} doping contents (Fig.3e).

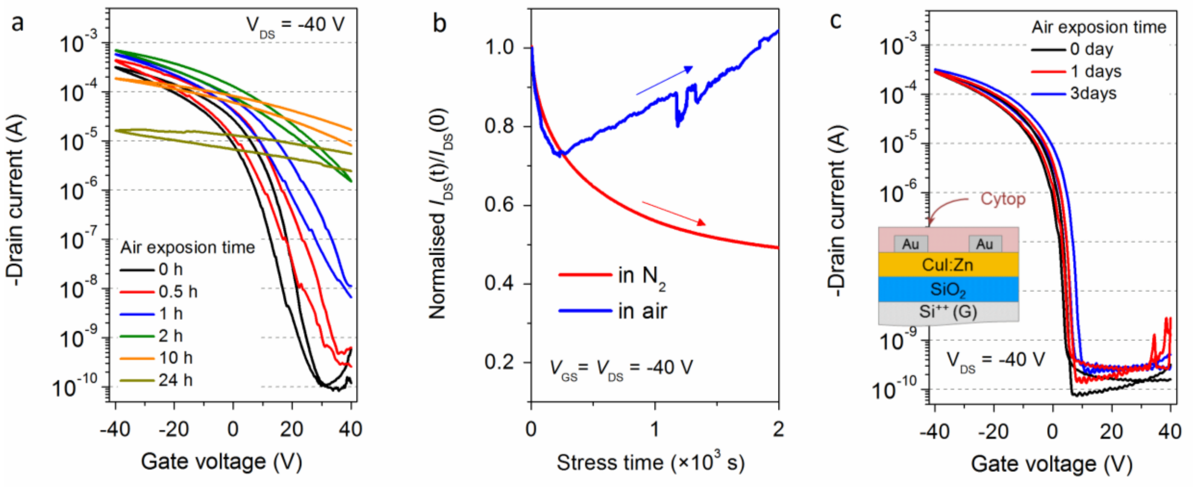

Supplementary Figure 22. (a) Transfer curves of the CuI:Zn TFT measured at a function of air exposition time (temperature = 25 °C; relative humidity ≈ 30%). (b) NBS-induced I_{DS} variations of the CuI:Zn TFTs in N_2 and air atmosphere. (c) Air stability behaviors of CuI:Zn TFT with CYTOP passivation layer. Cytop stoste was spin-coated on the top of the devices at 2000 rpm for 1 min (thickness: 1.2 μm). The pristine TFT μ_{sat} is 4.0 cm^2/Vs and V_{TH} is 5 V.

When the CuI:Zn TFT was exposed in air for short term, the obvious p-doping effect was observed with higher current level and right-shift threshold voltage. The p-doping originates from the absorption of O_2 from air. During the fabrication and annealing process of CuI:Zn thin films, the evaporation of a trace amount of iodine could occur, leaving iodide vacancies (electron donors) inside the films. Upon exposing the device in air, O_2 molecules can occupy iodide vacancies, resulting in acceptor-like electronic states.[5,6] During this period, the moisture can also diffuse into the crystal lattice of materials, resulting in the hole interaction with polar H_2O at grain boundaries. In addition, the adsorbed H_2O at grain boundaries can increase the energy barrier for hole transport.[7,8]

Due to the impressionable feature for CuI:Zn to O_2, the short-term explosion could lead to the p-doping phenomenon. Therefore, the I_{DS} showed abnormal increase under bias stress test in ambient condition.

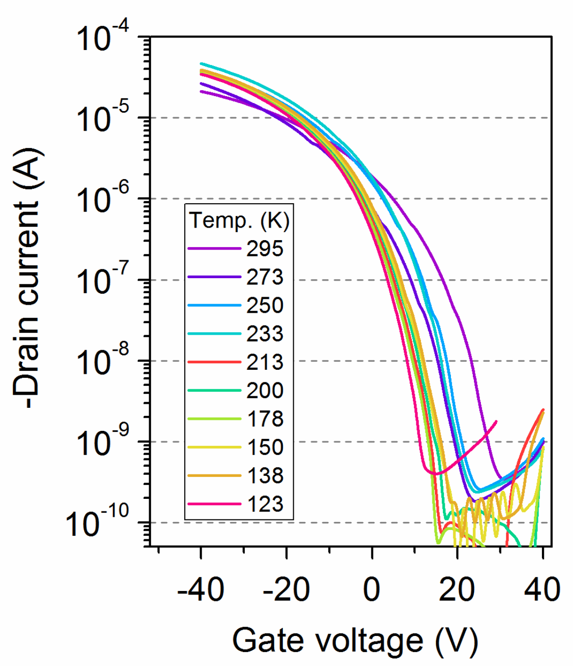

Supplementary Figure 23. Transfer curves of the CuI:Zn TFT measured at different temperatures (from 295 K to 123 K, V_{DS} = -1 V).

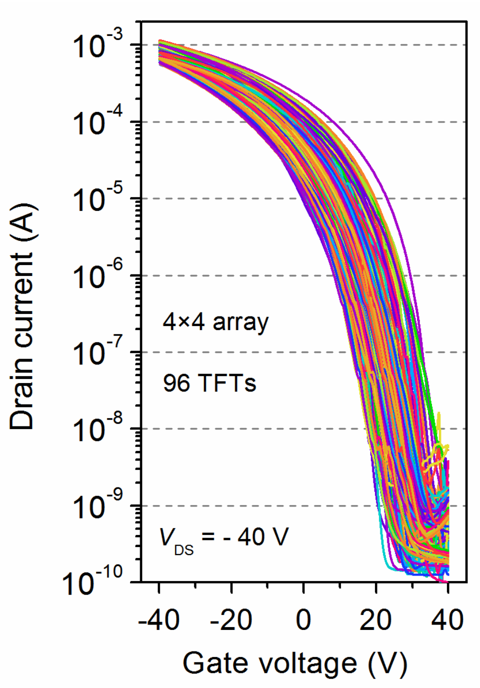

Supplementary Figure 24. Transfer characteristics of the wafer-scale CuI:Zn/SiO_2 TFTs (96 TFTs).

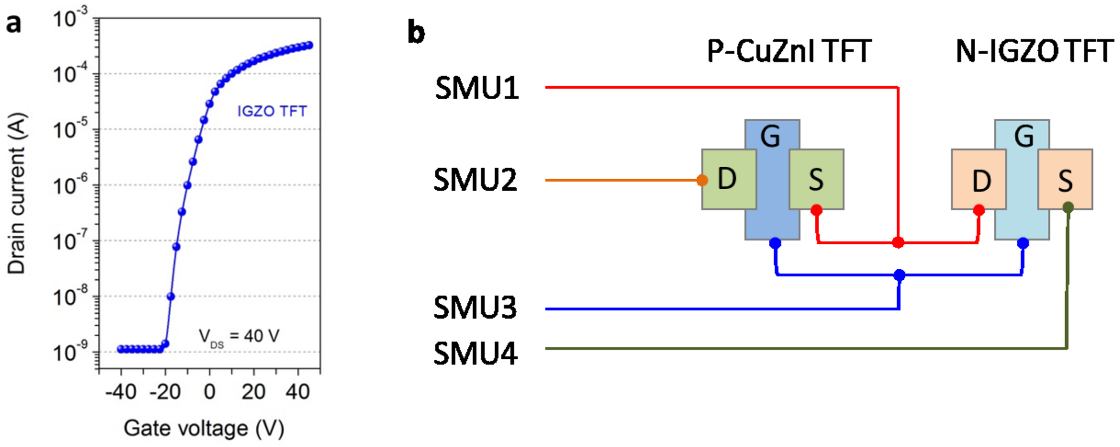

Supplementary Figure 25. (a) Transfer curve of the IGZO/SiO_2 TFT used for inverter fabrication. (b) Diagram description of inverter connection using cable.

The μ_{sat} and I_{on}/I_{off} of the n-channel IGZO TFT are 5.8 $cm^2\ V^{-1}\ s^{-1}$ and 3×10^5 (bottom-gate top-contact structure with 50-nm Al and 100-nm SiO_2 as source/drain electrodes and dielectric layer; channel width/length = 1000/300 μm). The experimental details for IGZO channel layer can be found in our previous report.[9]

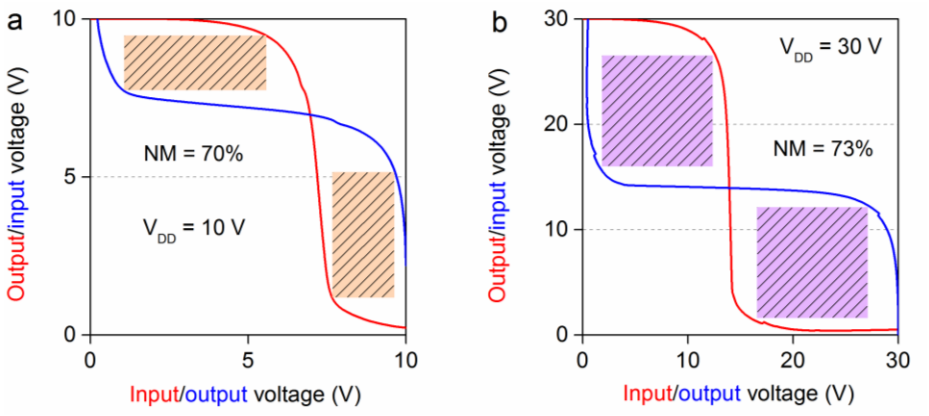

Supplementary Figure 26. Noise margin (NM) extraction from the butterfly plot at V_{DD} values of (a) 10 and (b) 30 V.

Supplementary Table 1. Carrier effective mass and band gaps for several known *p* and *n*-type oxide and (pseudo)halide semiconductors.

Material	Structure	Hole effective mass (m_h)<br>Electron effective mass (m_e)	Band gap (eV)	Ref.
Rh_2ZnO_4	spinel	$m_h \approx 7.00\ m_0$	2.74	[10]
$AlCuO_2$	delafossite	$m_h \approx 2.60\ m_0$	3.00	[11,12]
$SrCu_2O_2$	tetragonal	$m_h \approx 2.10\ m_0$	3.30	[13]
SnO	tetragonal	$m_h = 2.05\ m_0$	2.70 (direct)<br>0.70 (indirect)	[14,15]
Cu_2O	cubic	$m_h = 1.55\ m_0$	≈ 2.20	[16]
La_2SeO_2	--	$m_h = 0.92\ m_0$	3.49	[17]
CuSCN	hexagonal	$m_h = 0.80\ m_0$ (*c* axis)<br>$m_h = 0.50\ m_0$ (*ab* plane)	≈ 3.8	[18]
CuI	zincblende	$m_h = 0.30\ m_0$	3.1	[19]
In_2O_3	cubic	$m_e = 0.35\ m_0$	3.75	[20]

Supplementary Table 2. Key parameters of CuI TFTs doped with different dopants.

Component (thickness)	Doping ratio (mol%)	Saturation mobility [μ_{sat}] ($cm^2\ V^{-1}\ s^{-1}$)	On/off ratio [I_{on}/I_{off}]	Threshold voltage [V_{TH}] (V)
CuI:Zn (9 nm)	2.5	6.1 ± 0.5	(1-3) × 10^6	22.5 ± 3.5
	5	5.3 ± 0.3	7 × 10^6	17.5 ± 2.0
	10	2.0 ± 0.2	3 × 10^6	7.8 ± 1.0
	15	0.36 ± 0.04	~10^6	6.3 ± 0.5
CuI:Bi	2	1.7 ± 0.40	~10^2	--
	5	0.45 ± 0.05	(1–3) × 10^4	22.0 ± 2.0
	10	0.08 ± 0.02	~10^4	4.5 ± 0.5
CuI:Pb	2	0.80 ± 0.30	10^2~10^3	--
	5	0.34 ± 0.10	(1–3) × 10^4	24.5 ± 3.0
	10	0.05 ± 0.02	10^3~10^4	2.2 ± 0.3
CuI:Ga	10–30	TFTs always on (I_{on}/I_{off} <10)		
CuI:Ni	10–20	TFTs always on (I_{on}/I_{off} =10~10^2)		
CuI:Sn	5–20	Inactive (TFTs always off)		

Supplementary Table 3. Recent achievements of solution processed p-type oxide and (pseudo)halide TFTs on SiO_2 gate dielectric.

Method	Channel (thickness, nm)	Annealing temp. (°C)	Annealing time (h)	μ_{FE} ($cm^2 V^{-1} s^{-1}$)	I_{on}/I_{off}	Inverter gain (V/V)	Year	Ref.
Spin coating	SnO (14.7)	450	2.0	0.13	85	×	2012	21
Spin coating	Cu_2O (100)	700	1.5	0.16	~10^2	×	2013	22
Spray pyrolysis	Cu_2O (40)	275	12.0	10^{-4}–10^{-2}	4×10^3	×	2013	23
Spin coating	CuSCN (20)	80	0.25	10^{-3}–10^{-2}	~10^4	2	2013	24
Spin coating	NiO (40)	500	1.0	0.14	--	×	2014	25
Spin coating	Cu_2O (17.5)	600	>2.0	0.29	~10^4	×	2015	26
Spin coating	CuO (--)	500	1.0	0.01	~10^3	×	2016	27
Spin coating	NiO (--)	250	1.0	0.07	~10^4	×	2016	28
Ink-jet printing	CuI (100)	60	--	--	10~10^2	×	2016	29
Spin coating	CuO (12.2)	250	1.0	0.30	~10^4	×	2017	30
Spin coating	NiO (30)	350	>1.0	0.01	2	×	2017	31
Spin coating	CuO (--)	500	1.0	4×10^{-4}	~10^2	×	2018	32
Ink-jet printing	NiO (15)	280	>2.0	0.01	4×10^2	×	2018	33
Spin coating	CuSeCN (15)	140	0.3	0.002	~10^3	×	2018	34
Spin coating	$CuAlO_2$ (40)	1000	2.0	~0.1	~10^3	×	2018	35
Spin coating	CuO_x (20)	500	1.0	~10^{-3}	~10^4	×	2018	36
Spin coating	CuI (5)	RT	0	0.40	5×10^2	4	2018	[2]
Spin coating	CuO (--)	600	3.5	~10^{-3}	~10^3	×	2019	37
Spin coating	CuO (7)	220	1.0	0.15	~10^4	37	2019	38
Ink-jet printing	NiO (20)	175 (laser)	--	0.9	~10^5	10	2019	39
Spin coating	NiO (53)	250	1.0	0.48	~10^3	×	2019	40
Spin coating	CuI:$Zn_{5mol\%}$ (9)	80	0.16	5.3	7 × 10^6	56	this study	

Note: "--" means not mentioned in the literature; "×" means not demonstrated in the paper.

Supplementary References

1 Ramana, C. *et al.* X-ray photoelectron spectroscopy depth profiling of La_2O_3/Si thin films deposited by reactive magnetron sputtering. *ACS Appl. Mater. Interfaces* **3**, 4370-4373 (2011).

2 Liu, A. *et al.* Room-temperature solution-synthesized p-type copper(I) iodide semiconductors for transparent thin-film transistors and complementary electronics. *Adv. Mater.* **30**, 1802379 (2018).

3 Pei, K., Chen, M., Zhou, Z., Li, H. & Chan, P. K. L. Overestimation of Carrier Mobility in Organic Thin Film Transistors Due to Unaccounted Fringe Currents. *ACS Applied Electronic Materials* **1**, 379-388 (2019).

4 Park, J. H. *et al.* Low-Temperature, High-Performance Solution-Processed Thin-Film Transistors with Peroxo-Zirconium Oxide Dielectric. *ACS Appl Mater Interfaces* **5**, 410-417 (2013).

5 Szemjonov, A. *et al.* Impact of Oxygen on the Electronic Structure of Triple-Cation Halide Perovskites. *ACS Materials Letters* **1**, 506-510 (2019).

6 Inudo, S., Miyake, M. & Hirato, T. Electrical properties of CuI films prepared by spin coating. *physica status solidi (a)* **210**, 2395-2398 (2013).

7 Park, I.-J. *et al.* Bias-Stress-Induced Instabilities in P-Type Cu_2O Thin-Film Transistors. *IEEE Electron Device Lett* **34**, 647-649 (2013).

8 Han, Y.-J. *et al.* Improvement of long-term durability and bias stress stability in p-type SnO thin-film transistors using a SU-8 passivation layer. *Electron Device Lett,* **35**, 1260-1262 (2014).

9 Lee, W.-J. *et al.* Large-Scale Precise Printing of Ultrathin Sol-Gel Oxide Dielectrics for Directly Patterned Solution-Processed Metal Oxide Transistor Arrays. *Adv Mater* **27**, 5043-5048 (2015).

10 Nagaraja, A. R. *et al.* Band or Polaron: The Hole Conduction Mechanism in the p ‐ Type Spinel Rh_2ZnO_4. *J Am Ceram Soc* **95**, 269-274 (2012).

11 Pellicer-Porres, J. *et al.* On the band gap of $CuAlO_2$ delafossite. *Appl Phys Lett* **88**, 181904 (2006).

12 Hautier, G., Miglio, A., Ceder, G., Rignanese, G. M. & Gonze, X. Identification and design principles of low hole effective mass p-type transparent conducting oxides. *Nat Commun* **4**, 2292-2292 (2013).

13 Ohta, H. *et al.* Electronic structure and optical properties of $SrCu_2O_2$. *J Appl Phys* **91**, 3074-3078 (2002).

14 Ogo, Y. *et al.* Tin monoxide as an s-orbital-based p-type oxide semiconductor: Electronic structures and TFT application. *Physica Status Solidi (A)* **206**, 2187-2191 (2009).

15 Nomura, K., Kamiya, T. & Hosono, H. Ambipolar oxide thin-film transistor. *Adv Mater* **23**, 3431-3434 (2011).

16 Fortunato, E. *et al.* Thin-film transistors based on p-type Cu_2O thin films produced at room temperature. *Appl Phys Lett* **96**, 192102 (2010).

17 Sarmadian, N., Saniz, R., Partoens, B. & Lamoen, D. Easily doped p-type, low hole effective mass, transparent oxides. *Sci Rep* **6**, 20446 (2016).

18 Jaffe, J. E. *et al.* Electronic and defect structures of CuSCN. *J Phys Chem C* **114**, 9111-9117 (2010).

19 Grundmann, M. *et al.* Cuprous iodide–a p-type transparent semiconductor: history and novel applications. *physica status solidi (a)* **210**, 1671-1703 (2013).

20 Edwards, P. P., Porch, A., Jones, M. O., Morgan, D. V. & Perks, R. M. Basic materials physics of transparent conducting oxides. *Dalton Transactions*, 2995-3002 (2004).

21 Okamura, K., Nasr, B., Brand, R. A. & Hahn, H. Solution-processed oxide semiconductor SnO in p-channel thin-film transistors. *J Mater Chem* **22**, 4607 (2012).

22 Kim, S. Y. *et al.* P-channel oxide thin film transistors using solution-processed copper oxide. *ACS Appl Mater Interfaces* **5**, 2417-2421 (2013).

23 Pattanasattayavong, P., Thomas, S., Adamopoulos, G., McLachlan, M. A. & Anthopoulos, T. D. p-channel thin-film transistors based on spray-coated Cu_2O films. *Appl Phys Lett* **102**, 163505 (2013).

24 Pattanasattayavong, P. *et al.* Hole-Transporting Transistors and Circuits Based on the Transparent Inorganic Semiconductor Copper (I) Thiocyanate (CuSCN) Processed from Solution at Room Temperature. *Adv Mater* **25**, 1504-1509 (2013).

25 Liu, S. *et al.* Nickel Oxide Hole Injection/Transport Layers for Efficient Solution-Processed Organic Light-Emitting Diodes. *Chem Mater* **26**, 4528-4534 (2014).

26 Yu, J. *et al.* Solution-processed p-type copper oxide thin-film transistors fabricated by using a one-step vacuum annealing technique. *J Mater Chem C* **3**, 9509-9513 (2015).

27 Jang, J., Chung, S., Kang, H. & Subramanian, V. P-type CuO and Cu_2O transistors derived from a sol–gel copper (II) acetate monohydrate precursor. *Thin Solid Films* **600**, 157-161 (2016).

28 Liu, A. *et al.* Hole mobility modulation of solution-processed nickel oxide thin-film transistor based on high-k dielectric. *Appl Phys Lett* **108**, 233506 (2016).

29 Choi, C.-H. *et al.* Low-temperature, inkjet printed p-type copper(i) iodide thin film transistors. *J Mater Chem C* **4**, 10309-10314 (2016).

30 Liu, A. *et al.* In situ one-step synthesis of p-type copper oxide for low-temperature, solution-processed thin-film transistors. *J Mater Chem C* **5**, 2524-2530 (2017).

31 Li, Y., Liu, C., Wang, G. & Pei, Y. Investigation of solution combustion-processed nickel oxide p-channel thin film transistors. *Semicond Sci Tech* **32**, 085004 (2017).

32 Lee, S. *et al.* Sol-Gel Processed p-Type CuO Phototransistor for a Near-Infrared Sensor. *IEEE Electron Device Lett* **39**, 47-50 (2018).

33 Hu, H., Zhu, J., Chen, M., Guo, T. & Li, F. Inkjet-printed p-type nickel oxide thin-film transistor. *Appl Surf Sci* **441**, 295-302 (2018).

34 Wijeyasinghe, N. *et al.* Copper (I) Selenocyanate (CuSeCN) as a Novel Hole−Transport Layer for Transistors, Organic Solar Cells, and Light−Emitting Diodes. *Adv Funct Mater* **0**, 1707319.

35 Li, S. *et al.* Preparation and Characterization of Solution-Processed Nanocrystalline p-Type $CuAlO_2$ Thin-Film Transistors. *Nanoscale research letters* **13**, 259 (2018).

36 Jung, T. S. *et al.* Enhancement of Switching Characteristic for p-Type Oxide Semiconductors Using Hypochlorous Acid. *ACS Appl Mater Interfaces* **10**, 32337-32343 (2018).

37 Lee, H., Zhang, X., Kim, E.-J. & Park, J. Structural and Electrical Characteristics of Solution-processed Copper Oxide Films for Application in Thin-film Transistors. *Sensor Mater* **31**, 501-507 (2019).

38 Liu, A., Zhu, H. & Noh, Y.-Y. Polyol Reduction: A Low-Temperature Eco-Friendly Solution Process for p-Channel Copper Oxide-Based Transistors and Inverter Circuits. *ACS Appl Mater Interfaces* **11**, 33157-33164 (2019).

39 Chen, C., Yang, Q., Chen, G., Chen, H. & Guo, T. Solution-Processed Oxide Complementary Inverter via Laser Annealing and Inkjet Printing. *Ieee T Electron Dev* **66**, 4888-4893 (2019).

40 Xu, W. *et al.* p-Type transparent amorphous oxide thin-film transistors using low-temperature solution-processed nickel oxide. *J Alloy Compd* **806**, 40-51 (2019).
